# Rural Hospital Administrators’ Beliefs About Safety, Financial Viability, and Community Need for Offering Obstetric Care

**DOI:** 10.1001/jamahealthforum.2022.0204

**Published:** 2022-03-25

**Authors:** Katy B. Kozhimannil, Julia D. Interrante, Lindsay K. Admon, Bridget L. Basile Ibrahim

**Affiliations:** 1 University of Minnesota Rural Health Research Center, Division of Health Policy and Management, University of Minnesota School of Public Health, Minneapolis; 2 Department of Obstetrics and Gynecology, University of Michigan, Ann Arbor; 3 Institute for Healthcare Policy and Innovation, University of Michigan, Ann Arbor

## Abstract

**Importance:**

Rural obstetric unit closures are associated with adverse maternal and infant health outcomes and are most common among low–birth volume facilities located in remote areas. Declining access to obstetric care is a concern in rural communities in the US.

**Objective:**

To assess rural hospital administrators’ beliefs about safety, financial viability, and community need for offering obstetric care.

**Design, Setting, and Participants:**

Using the American Hospital Association Annual Survey to identify rural hospitals with obstetric units, we developed and conducted a national survey of a sample of rural hospitals that provided obstetric services in 2021. Obstetric unit managers or administrators at 292 rural hospitals providing obstetric services were surveyed from March to August 2021.

**Exposures:**

Local factors, clinical safety, workforce, and financial considerations for obstetric services at participating hospitals.

**Main Outcomes and Measures:**

Hospital-level decisions on maintaining obstetric care.

**Results:**

Of the 93 total responding hospitals (32% response rate), 33 (35.5%) were critical access hospitals, 60 (64.5%) were located in micropolitan rural counties; they had a median (IQR) average daily census of 22 (10-53) patients, and 48 (52.2%) had experienced a recent decline in births, with a median (IQR) of 274 (120-446) births in 2019. Respondents reported that the minimum number of annual births needed to safely provide obstetric care was 200 (IQR, 100-350). From a financial perspective, the minimum number of annual births needed was also 200 (IQR, 120-360). When making decisions about maintaining obstetric care, 51 (64.6%) responding hospitals listed their highest priority as meeting local community needs, 13 (16.5%) listed financial considerations, and 10 (12.7%) listed staffing. Overall, 23 (25%) responding hospitals were not sure they would continue providing obstetrics, or they expected to stop offering this service.

**Conclusions and Relevance:**

In this survey of US rural hospitals that offer obstetric services, many administrators indicated prioritizing local community needs for obstetric care over concerns about financial viability and staffing.

## Key Points

**Question:**

What are rural hospital administrators’ beliefs about safety, financial viability, and community need for offering obstetric care?

**Findings:**

In this survey of US rural hospitals providing obstetric care, administrators reported needing at least 200 annual births for safety and financial viability. Local maternity care needs strongly influenced hospital decisions to maintain obstetric services, even below that threshold; 1 in 4 surveyed hospitals were unsure if they would continue providing obstetric services.

**Meaning:**

Policies to improve rural obstetric care access should account for administrative concerns about safety, workforce, financial viability, and community needs.

## Teaser

This survey study examines rural hospital administrators’ beliefs about safety, financial viability, and community need for offering obstetric at 292 rural hospitals.

## Introduction

Rural residents face barriers to accessing high-quality health care at all life stages, but particularly so before, during, and after pregnancy.^1,2^ Maternal and infant health challenges faced by families in rural US communities are a serious concern, and both maternal and infant morbidity and mortality are higher among rural compared with urban families.^3,4^ Rural hospitals are closing, and rural communities are losing access to health care services locally, including obstetrics.^5,6^ In 2014, more than half of rural counties lacked hospital-based obstetric care.^7^ Since 2014, the trend of rural obstetric unit closures has continued, disproportionately affecting remote (not adjacent to urban counties) and less populated rural counties.^8^ By 2018, only 40% of rural counties had a hospital that provided inpatient obstetric care.^8^ The consequences of losing these services in more remote rural counties included reductions in prenatal care and increases in preterm birth, births in emergency departments, out-of-hospital birth, and cesarean births.^9^

Safely maintaining access to obstetric care in rural communities is a priority. Prior research and stakeholder input indicate 4 main challenges rural hospitals face in providing inpatient obstetric services: local community factors (distance to the nearest hospital with tertiary care, patient complexity, availability of medical transportation), clinical safety (maintaining basic skills, ensuring access to necessary equipment and specialized expertise for high-risk clinical conditions), workforce (clinician scheduling, recruitment, and retention), and financial considerations (high fixed costs, reimbursement rates, payer mix, hospital ownership structure, and costs of malpractice insurance).^10,11^

There have been broad clinical efforts to create protocols and resources to support safety in obstetric care.^12,13,14^ These include the Alliance for Innovation on Maternal Health (AIM) program, which has created a set of patient safety care bundles to address preventable severe maternal morbidity and mortality,^15,16,17^ and the California Maternal Quality Care Collaborative (CMQCC), which uses research, quality improvement toolkits, and state-wide outreach collaboratives to improve maternal and infant health.^18,19^ Current clinical and policy initiatives aimed at improving rural maternity care access are hampered by a lack of comprehensive evidence from rural settings about the particular challenges and trade-offs in their communities and health care systems.^20,21^ To address this, we conducted a national survey of a sample of rural hospitals to describe their perspectives on criteria for safe provision of obstetric care in rural US settings.

## Methods

This research was reviewed by the University of Minnesota institutional review board and designated as exempt because all data used were deidentified. We followed the American Association for Public Opinion Research Standard Definition guidelines for reporting findings from this survey.

### Data and Sampling Frame

We used American Hospital Association (AHA) Annual Survey data from 2010 to 2018 to create the sampling frame. We identified all short-term acute care hospitals located in counties in nonmetropolitan statistical areas (micropolitan and noncore).^22^ Then we identified hospital obstetric service provision status using a previously described multistep approach.^7,23^ Because of racial inequities in maternal and infant outcomes,^24,25^ we surveyed all hospitals with obstetric services in rural counties where most residents are Black and Indigenous people, or racial and ethnic minorities, and created a random sample of 20% of hospitals with obstetric services in rural counties with a majority-White population. County-level demographics on race and ethnicity were drawn from the US Census, 2010 and were grouped as majority-White vs majority racial and ethnic minorities–based, consistent with prior research on rural inequities.^26^ After removing hospitals that closed and reclassifying hospitals based on their survey-reported obstetric provision status, we included 292 rural hospitals providing obstetric services in the sample (194 in majority-White rural counties and 98 in racial and ethnic minorities majority rural counties).^23^

### Survey Development

We developed the Safe Maternity Care survey instrument with input from rural clinicians and hospital administrators and members of a rural health expert work group. The online survey was built and administered in the Qualtrics platform (Provo, Utah). The survey was piloted with administrators at 6 rural hospitals, and questions were edited for clarity. The final survey instrument (included as eMethods in the Supplement) consisted of 47 questions organized into 4 topic areas: local factors, training and clinical safety, workforce and staffing, and finances. For each topic area, there were 1 or 2 open-ended questions designed to elucidate more information from respondents, including the following: “Please explain how you decided on the number of births/clinicians necessary,” and “Please explain your answer above.”

### Data Collection

At initiation of data collection, we contacted hospital chief executive officers (CEO) and chief nursing officers (CNO) via email to describe the purpose of the study. For each hospital, we included a link to our survey and requested that the CEO/CNO forward the link to the nurse manager of the obstetric unit or another person best placed to answer questions about obstetric services. We sent 2 reminder emails to the CEO/CNO. After 6 weeks, the study team began telephoning hospitals and offered respondents the opportunity to provide responses by phone or by an emailed link to complete the survey online. After 3 months, we mailed postcards to the nurse manager of the obstetric units for hospitals who had not yet responded. The survey was open for data collection from March to August 2021. We received responses from 32% of hospitals in the study sample. Administrators from all but 1 hospital completed the survey online; 1 completed the survey by phone with study staff. Characteristics of responding hospitals and nonresponding hospitals were similar and are shown in eTable 1 in the Supplement.

### Analysis

We conducted a descriptive analysis. Categorical variables were reported as frequency and percent, and continuous variables were described using median and interquartile range (IQR). All analyses were conducted using SAS statistical software (version 9.4, SAS Institute).

## Results

Ninety-three hospital or obstetric unit administrators responded to the survey on behalf of their hospitals. Sociodemographic characteristics of the respondents are provided in eTable 2 in the Supplement. Key characteristics of responding hospitals are described in Table 1. Among respondents, 33 (35.5%) were Critical Access Hospitals, 60 (64.5%) were in micropolitan rural counties, whereas the other 33 (35.5%) were in noncore rural counties, distributed across all US regions (5 [5.4%] in the Northeast, 29 [31.2%] in the Midwest, 26 [27.9%] in the South, 33 [35.5%] in the West). Responding hospitals were generally small, with a median (IQR) average daily census of 22 (10-53) total patients, and most experienced a decline in annual births over the prior 3 years (48 [52.2%]; only 12 [13.0%] experienced an increase in births over that period), referred fewer than 10% of patients out of their hospital to a higher level of care (50 [55.6%]), and had the closest neonatal intensive care unit (NICU) over 60 miles away (50 [56.2%]; only 5 [5.6%] had an on-site NICU). Respondents were asked to predict whether they will be providing obstetric services at their hospital in 10 years, assuming no major changes in local community needs, workforce, or obstetric care financing. Some were unsure whether they would still be offering obstetric care in 10 years (19 [20.4%]), or even more dire, predicted that they were likely to close their obstetric unit (4 [4.3%]).

**Table 1.** Descriptive Statistics of 93 Rural Hospitals in the Study Sample^a^

Characteristic	No. (%)
Financial structure	
Hospital type	
Critical access hospitals	33 (35.5)
Location	
County type	
Micropolitan	60 (64.5)
Noncore	33 (35.5)
Region	
Northeast	5 (5.4)
Midwest	29 (31.2)
South	26 (27.9)
West	33 (35.5)
County racial demographics	
Majority racial and ethnic minorities	29 (31.2)
Hospital size	
Average daily census, median (IQR)	22 (10-53)
Obstetric services	
Change in annual births in last 3 y	
Increased	12 (13.0)
Decreased	48 (52.2)
Stayed the same	32 (34.8)
Pregnant patients referred out of hospital to higher level of care, %	
<10	50 (55.6)
10-24	33 (36.7)
>25	2 (2.2)
Distance to closest NICU	
On-site	5 (5.6)
10-29 Miles	5 (5.6)
30-60 Miles	29 (32.6)
>60 Miles	50 (56.2)
Prediction about whether hospital will continue to provide obstetrics in 10 y	
Confident yes	70 (75.2)
Unsure/too difficult to predict	19 (20.4)
Likely to close	4 (4.3)

Abbreviation: NICU, neonatal intensive care unit.

^a^ Not all categories add up to the total sample because of responses of “don’t know” or “missing.”

Hospitals reported a median (IQR) of 274 (120-446) births in 2019 (Table 2). Respondents reported that a median (IQR) of 200 (100-359) annual births were necessary to ensure their clinicians had enough experience to provide obstetric care safely. Hospitals that provided an estimate (n = 49) indicated that a median (IQR) of 200 (120-360) births annually were needed to make provision of inpatient obstetric services financially viable, though not necessarily profitable. Notably, only 49 of 93 responding hospitals provided an estimate of the minimum number of births needed for financial viability; it is possible that many respondents in clinical administration may not be involved in financial discussions and thus were not able to estimate this. At an individual hospital level, 29.9% (26 of 87 responding to both questions) reported having fewer actual births than they reported needing for clinical safety, whereas 41.7% (20 of 48 responding to both questions) reported having fewer actual births than they reported needing for financial viability. When asked an open-ended question to describe how they determined the minimum numbers of births needed for safety and financial viability, respondents described historic trends, financial breakeven points, and compliance with minimum nurse staffing guidelines. As 1 respondent noted, “we have seen a steady decline in obstetrics business over the years. It is not a profitable endeavor, and this is the number at which we would most likely begin serious discussions on viability.” Another respondent noted that obstetrics is “not a money maker.”

**Table 2.** Minimum Criteria for Rural Obstetric Services Provision: Safety and Finances^a^

Criteria	Responding, No.	Median (IQR)
Births needed to provide obstetric care safely	89	200 (100-350)
Births needed to make obstetric services financially viable	49	200 (120-360)
Actual births in 2019	91	274 (120-446)
Respondents that reported fewer actual births than		
Births needed to provide obstetric care safely, No. (%)	87	26 (29.9)
Births needed to make obstetric services financially viable, No. (%)	48	20 (41.7)

^a^ Not all categories add up to the total sample because of responses of “don’t know.”

Table 3 provides information about clinical safety in rural hospitals that provide obstetric care. Eighty-six (97%) responding hospitals noted that they had both training and equipment available to provide blood transfusions, neonatal resuscitation, and an emergency cesarean delivery within 1 hour. There was a split among hospitals, with 41 (46.6%) having an operating room that was dedicated for obstetric patients requiring surgical birth, and 45 (51.1%) having a general operating room, shared across the hospital’s units. Two (2%) responding hospitals said that they did not provide cesarean deliveries. Nearly half (42 [47.7%]) of responding hospitals reported using CMQCC toolkits and AIM patient safety bundles as resources to reduce the risk of maternal morbidity and mortality; others used solely the AIM bundles (18 [20.5%]) or CMQCC toolkits (12 [13.6%]). However, 16 (18.2%) respondents did not use either of these quality improvement resources.

**Table 3.** Components of Clinical Safety in 88 Rural Hospital Obstetric Units^a^

Clinical safety component	No. (%)
Training/equipment to provide	
Blood transfusions	87 (98.9)
Neonatal resuscitation	88 (100)
Emergency cesarean within 1 hour	86 (97.7)
Operating room for cesarean deliveries	
Dedicated for obstetric cases	41 (46.6)
Main/general operating room	45 (51.1)
Do not provide cesarean delivery	2 (2.3)
Resources to reduce maternal mortality and morbidity	
CMQCC toolkits only	12 (13.6)
AIM patient safety bundles only	18 (20.5)
Both CMQCC and AIM	42 (47.7)
Neither	16 (18.2)

Abbreviations: AIM, Alliance for Innovation on Maternal Health; CMQCC, California Maternal Quality Care Collaborative.

^a^ Five respondents did not answer these questions.

Responding hospitals ranked the influence of the following 4 factors on the hospital’s decision to continue to provide inpatient obstetric care: local community needs, clinical safety and training, staffing, and financial issues (Figure). For most, the most influential factor (ranked first) was local community needs (64.6%). In replying to an optional open-ended survey question asking for further explanation, 1 survey respondent said, “Many of the people who live here are poor and do not have vehicles to go elsewhere. They would come up here to deliver [babies] even if we did not have an obstetrics department.” Financial factors were most influential for 16.5%, staffing for 12.7%, and clinical safety and training for 6.3% of hospitals. Clinical safety and staffing were ranked second or third for a large portion of respondents, whereas financial considerations were ranked fourth by 62.0%.

**Figure.**
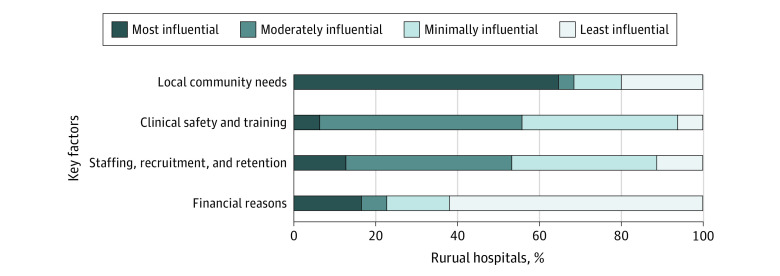
Ranking the Influence of Key Factors on 79 Rural Hospitals’ Decisions to Continue to Provide Inpatient Obstetric Care^a^ ^a^Fourteen respondents did not answer these questions.

## Discussion

Policy and clinical efforts to address the US maternal health crisis, and especially the geographic manifestations of the crisis, require data from rural communities on the challenges and constraints they face, including the criteria hospital administrators deem necessary for safely providing obstetric services. The rural hospitals we surveyed provided this information. With a median (IQR) of 274 (120-446) births per year, responding hospitals reported that the minimum annual number of births they needed to safely provide maternity services was 200 (IQR, 100-350). Twenty-six (30%) responding hospitals reported having fewer actual births than they reported were necessary to safely offer care. Results from this survey indicated that some rural hospital and unit administrators were worried about safely meeting the clinical care needs of local residents who are pregnant and giving birth.

Clinical safety is not the only consideration mentioned by rural hospital administrators regarding obstetric services. Hospitals have long reported financial constraints as a barrier to providing obstetric care or as a pressure for obstetric units closure.^7,10^ In this survey, 20 (41.7%) hospitals reported having fewer actual births than they reported needing for their obstetric service line to remain financially viable. As with clinical safety, rural hospitals reported that they would need a median (IQR) of 200 (120-360) births for financial viability of their obstetric unit. Several respondents noted that they viewed obstetrics as an essential community service that they plan to continue even if it is not financially beneficial to the hospital or health care system.

In 2018, across the entire US, there were 632 rural hospitals that had fewer than 200 births, according to AHA survey data; this is approximately 36% of all rural hospitals with obstetric services. Each of these may be at risk for falling below what the hospital administrators or obstetric unit leaders feel is a threshold for having the requisite experience and resources to provide safe, high-quality inpatient care for labor and delivery. Yet, many of these hospitals keep their obstetric units open. Our analysis points to several potential reasons. When making decisions about obstetric service lines, 51 (64.6%) responding hospitals prioritized local community needs as their top concern. This implies that rural hospital administrators feel that local conditions require obstetric services be available, even when the birth volume does not reach a threshold that the hospital knows is viable for safety or finances, because residents need a place to give birth locally. That is, local needs matter to rural hospital decision makers, and rural hospitals may require more financial and human resources to meet local needs.

Importantly, rural hospital and obstetric unit administrators from 23 (25%) responding hospitals revealed that they are not certain whether they will be able to continue to provide this service. There are fewer births in rural hospitals every year owing to declining overall US birth rates as well as rural residents giving birth at urban hospitals.^27,28^ Four (4%) responding rural hospitals predicted they would likely close their obstetric unit in the coming years, which is consistent with recent trends in rural obstetric unit closures and hospital consolidations.^7,29,30^ Additional closures, on top of decades of declining access, could extend the nearest obstetric unit further and further away for rural residents.^31^ One in every 5 rural residents is a reproductive-age woman, in rural counties both with and without hospital-based obstetric care, and rural residents will need a place to give birth.^32^

Rural hospital administrators indicate a commitment to retaining obstetric services whenever possible. Respondents were aware and wished to avoid potential consequences of obstetric unit closures, including adverse maternal and infant health outcomes, more births occurring in hospital emergency departments, and potential financial losses to rural communities that are already suffering economically.^9,13,33^ Findings from this analysis indicate that most rural hospitals take advantage of resources designed to help hospitals address maternal morbidity and mortality, 72 (80%) rural hospitals surveyed reported using the CMQCC toolkits and/or AIM patient safety bundles in their obstetric units, further demonstrating the commitment rural hospitals make to providing safe obstetric care in their communities.

For rural hospitals that must close their obstetric units, but continue to operate an emergency department, it is important to recognize that births may still occur at these hospitals. Indeed, a recent survey of rural emergency department administrators showed that 28% of rural hospitals without obstetric units had births occur in their emergency department; of these, 32% had unanticipated adverse birth outcomes, 22% experienced a delay in urgent transport, and 80% reported a need for additional training and/or resources for emergency obstetrics, which could include neonatal resuscitation, precipitous childbirth, and management of serious complications such as postpartum hemorrhage.^34,35^

To support rural hospitals in maintaining sufficient birth volume and clinical capacity to safely provide obstetric services, attention to financing of maternity care ought to include discussion of low-volume adjustments. Medicaid programs fund more than half of rural births, thus Medicaid payment policies and reimbursement rates play a key role in financial viability for rural obstetric care, as Medicaid pays substantially lower rates for childbirth, compared with private insurance.^21,36,37^ Pregnant Medicaid beneficiaries living in rural areas are less likely than the privately insured to give birth at an urban hospital or at a hospital with neonatal intensive care, even after controlling for clinical conditions that may require higher acuity services.^27,38^ Payment reform may help to support local childbirth for rural residents when it is safe, and efficient referral and transfer for all who need higher levels of care or services provided in urban areas.

Rural representation in policy decision-making bodies, from federal to state programs to clinical and professional associations to maternal mortality review committees, is crucial for providing the distinct perspective of rural residents, communities, and health care delivery systems.^39^ Ensuring a continued rural presence and voice in widely-regarded and successful national and state efforts to improve maternal and infant health, including CMQCC and AIM, will help to ensure that these tools and resources are relevant and useful in rural settings.

### Limitations

We hypothesize that the survey response rate was limited owing to strained staff and competing priorities during the COVID-19 pandemic. Nearly a third of hospitals included in the sample responded to the survey, and the respondents were broadly similar across almost all characteristics (eTable 1 in the Supplement), except that they were more likely to be located in the Western US and less likely to be located in the South, compared with nonresponding hospitals. In addition to potential effect on the response rate, the COVID-19 pandemic has put financial pressure on rural hospitals and may have affected responses about safety and financing. Although we designed a stratified survey to compare outcomes based on the racial demographics of rural communities, we did not have sufficient sample size to conduct stratified analyses owing to the response rate. Sample size limits the precision of estimates.

Item response for the question about the number of births necessary for financial viability was lower than for other main study questions, and may reflect a more limited perspective on finances for many survey respondents, compared with other hospital administrators, such as the CEO. Hospitals that do not provide obstetric services were not included in this study; therefore study findings could be biased downward toward a smaller number of necessary births. Finally, this analysis offers a point-in-time estimate of obstetric care and relies on respondent knowledge and recall, and responses are subject to social desirability bias. Still, these data provide the first national data, from a hospital perspective, on what is needed to offer safe obstetric care in rural communities. These data are sorely needed to inform policies aimed to improve access to obstetric care for rural residents and address maternal morbidity and mortality in communities across the US.

## Conclusions

In this survey of US rural hospitals that offer obstetric services, many administrators indicated prioritizing local needs for pregnancy and childbirth care over concerns about financial viability and staffing. Policies to improve rural obstetric care access should account for administrative concerns about community needs, clinical safety, and recruitment, retention, and training for the physician and nursing workforce. In addition, adjusting reimbursement policies for low-volume rural hospitals may help ensure financial viability and continued operations of rural hospitals’ obstetric service lines. Local obstetric care access is important for rural residents, and rural hospital administrators are committed to efforts to improve maternal and infant health in communities that already experience poor outcomes.

## Notes

Supplement. eMethods. Safe Maternity Care for All Rural Residents Survey eTable 1. Comparing Responding vs Nonresponding Hospitals Among Those With Current Obstetric Services eTable 2. Descriptive Information About Survey Respondents (n=93)
