# Supplementary material for: Rural Hospital Administrators’ Beliefs About Safety, Financial Viability, and Community Need for Offering Obstetric Care
Source: JAMA Health Forum. 2022 Mar 25;3(3):e220204. doi: 10.1001/jamahealthforum.2022.0204 (PMC8956977; doi:10.1001/jamahealthforum.2022.0204)
Supplement: Supplement. — eMethods. Safe Maternity Care for All Rural Residents Survey eTable 1. Comparing Responding vs Nonresponding Hospitals Among Those With Current Obstetric Services eTable 2. Descriptive Information About Survey Respondents (n=93) [file jamahealthforum-e220204-s001.pdf]

Supplemental Online Content

Kozhimannil KB, Interrante JD, Admon LK, Basile Ibrahim BL. Rural hospital administrators' beliefs about safety, financial viability, and community need for offering obstetric care. *JAMA Health Forum*. 2022;3(3):e220204.
doi:10.1001/jamahealthforum.2022.0204

eMethods. Safe Maternity Care for All Rural Residents Survey

eTable 1. Comparing Responding vs Nonresponding Hospitals Among Those With Current Obstetric Services

eTable 2. Descriptive Information About Survey Respondents (n=93)

This supplemental material has been provided by the authors to give readers additional information about their work.

© 2022 Kozhimannil KB et al. *JAMA Health Forum.*

eMethods: Safe Maternity Care for All Rural Residents Survey

Thank you very much for participating in our survey. If you have any questions or feedback, please contact the University of Minnesota's Rural Maternity Care Research Team at rmc@umn.edu.
Please provide us with your contact info. This information is for survey tracking and follow-up purposes. It will be kept confidential, and will not be associated with your answers to the survey in reporting.

Your Name ____
Your Phone ____
Your Email ____
Hospital Name ____
Hospital Street ____
Hospital City ____
Hospital State
▼ Alphabetical list of states dropdown [Alabama to Wyoming]
Hospital zip code____

If you know it, please provide your hospital's 6-digit CMS Certification Number (CCN) and/or American Hospital Association (AHA) ID number.
CMS Certification Number/ Hospital Medicare Provider Number
American Hospital Association ID

What is your highest level of education?
MD
PhD/DNP/Other doctorate
Master's degree
4 year degree
2 year degree
Some college
High school graduate

How many years have you worked at your current institution?
0-5 years
6-10 years
11-20 years
> 20 years (4)

What is your position or title?
Nurse Manager, Labor and Birth Unit
CEO
Chief Nursing Officer
Chief Medical Officer
Medical Director
Head/Chief of Obstetrics
Other, please explain ____

How did you initially hear about this survey?
Survey information email was passed along from someone in my hospital
Survey information was emailed directly to me
I spoke with someone on the telephone about the survey
I received a postcard with the link
Other, please explain ____

© 2022 Kozhimannil KB et al. *JAMA Health Forum.*

Does your hospital currently provide **inpatient** services for labor, birth, and postpartum care?
Yes
No

Skip To: Q10 If Does your hospital currently provide inpatient services for labor, birth, and postpartum care? = No
Skip To: End of Block If Does your hospital currently provide inpatient services for labor, birth, and postpartum care? = Yes

Start of Block: Questions for Hospitals that Currently Offer Inpatient Maternity Care Services

Our three main questions about rural inpatient maternity care are asked first and the remainder of the survey is divided into five sections: 1) Local Community, 2) Clinical Safety, 3) Staffing, 4) Finances, and 5) Wrap Up.

We estimate that this survey should take less than 30 minutes to complete. If you have questions or would like to provide feedback, please reach out to the study team at rmc@umn.edu. Thank you for participating!

Main question 1
Approximately how many births need to occur each year in your hospital to allow your clinicians (physicians, midwives, and nurses) enough experience to provide care safely?

____

Please explain how you decided on the number of births necessary.

____
____

Main question 2
What is the minimum number of maternity care providers (physicians who deliver babies or midwives) your hospital needs to provide inpatient labor and birth services?

	Minimum FTE
Obstetrician	_____
Family practice doctor	_____
Certified nurse midwife (CNM)	_____

Please explain how you decided on the number of providers necessary.

____
____

Main question 3
How many births per year do you need at your hospital to make provision of inpatient maternity services financially viable (**not necessarily profitable, but viable**)? (Please try to give an estimate to the best of your knowledge. If you can't provide a number, please enter 999.)

____

Please explain to help us understand how you arrived at this number.

____
____

Thank you for answering our main questions. We will now move on to Section 1: Local Community

Section 1: Local Community
This section will ask questions about the community where your hospital is located and the patients who give birth at your hospital.

© 2022 Kozhimannil KB et al. *JAMA Health Forum.*

Approximately how many births occurred in your hospital in **2019**? (If you don't know, please enter 9999)

____

Has the annual number of births changed in the last 3 years?
Increased
Decreased
Stayed about the same
I don't know

Do you anticipate the number of births will change in the next 3 years?
Births will likely increase
Births will likely decrease
Birth numbers will probably stay about the same
I can't say

Approximately how many live births occur in your **primary service area** each year? (This is the number you would use to calculate your hospital's market-share. If you don't know, please enter 9999)

____

Does your unit regularly provide labor and birth care for patients who are:

	Most of our patients (more than 50%)	Many of our patients (25-50%)	Some of our patients (10-24%)	A few of our patients (less than 10%)	Never	I don't know
Black or African American	○	○	○	○	○	○
Asian	○	○	○	○	○	○
Latinx or Hispanic	○	○	○	○	○	○
American Indian or Alaska Native	○	○	○	○	○	○
Migrant workers	○	○	○	○	○	○
Incarcerated	○	○	○	○	○	○
Refugees or recent immigrants	○	○	○	○	○	○

© 2022 Kozhimannil KB et al. *JAMA Health Forum.*

Which of the following are primary languages commonly spoken by patients who give birth at your hospital?

	Most of our patients (more than 50%)	Many of our patients (25-50%)	Some of our patients (10-24%)	A few of our patients (less than 10%)	None	I don't know
English	○	○	○	○	○	○
Spanish	○	○	○	○	○	○
Other	○	○	○	○	○	○

Display This Question:
If Which of the following are primary languages commonly spoken by patients who give birth at your h... = Other [Many of our patients (25-50%)]
Or Which of the following are primary languages commonly spoken by patients who give birth at your h... = Other [Most of our patients (more than 50%)]

What other primary language(s) are commonly spoken by your patients?

____

Display This Question:
If Which of the following are primary languages commonly spoken by patients who give birth at your h... = Spanish [Many of our patients (25-50%)]
Or Which of the following are primary languages commonly spoken by patients who give birth at your h... = Spanish [Most of our patients (more than 50%)]
Or Which of the following are primary languages commonly spoken by patients who give birth at your h... = Other [Many of our patients (25-50%)]
Or Which of the following are primary languages commonly spoken by patients who give birth at your h... = Other [Most of our patients (more than 50%)]

Do you have staff fluent in the common languages other than English you indicated above to provide appropriate care for birthing patients?
Yes
No
Other, please explain ____

© 2022 Kozhimannil KB et al. *JAMA Health Forum.*

Approximately how many of your labor and birth patients travel the following distances to get to your hospital for childbirth?

	Most of our patients (more than 50%)	Many of our patients (25-50%)	Some of our patients (10-24%)	A few of our patients (less than 10%)	None	I don't know
< 10 miles	○	○	○	○	○	○
10-29 miles	○	○	○	○	○	○
30-60 miles	○	○	○	○	○	○
> 60 miles	○	○	○	○	○	○

How far from your hospital is the next-nearest hospital that has labor and birth services?
< 10 miles
10-29 miles
30-60 miles
> 60 miles
I don't know

What percentage of pregnant patients do you refer out of your hospital to a higher level of care for delivery?
< 10%
10-24%
25-50%
> 50%
I don't know

Which of the following options for maternity care are available through your hospital and/or in your community?

	Availability			
	In or affiliated with my hospital	In the community but not affiliated with my hospital	Not available locally	I don't know
Individual prenatal care	○	○	○	○
Group prenatal care	○	○	○	○
Midwifery care with certified nurse midwives (CNM)	○	○	○	○

© 2022 Kozhimannil KB et al. *JAMA Health Forum.*

Which of the following resources are available through your hospital and/or in your community?

	Availability			
	In or affiliated with my hospital	In the community but not affiliated with my hospital	Not available locally	I don't know
Childbirth education classes	○	○	○	○
Nutrition/WIC program	○	○	○	○
Perinatal mental health services	○	○	○	○
Doula care	○	○	○	○
Postpartum support groups	○	○	○	○
Breastfeeding support groups	○	○	○	○
Nurse home visiting for prenatal patients	○	○	○	○
Nurse home visiting for postpartum patients	○	○	○	○

Which of the following are available to patients who give birth in your hospital?

	Offered in your hospital	
	Yes	No
Epidural anesthesia for labor	○	○
Birthing tub	○	○
Vaginal birth after cesarean (VBAC)	○	○
Lactation support from nursing staff	○	○
Lactation support from dedicated board-certified lactation consultants (IBCLC)	○	○

© 2022 Kozhimannil KB et al. *JAMA Health Forum.*

What is your bypass rate? i.e. What percentage of local pregnant patients choose to give birth at non-local hospitals (more than 30 minutes away) **for nonmedical reasons**?
< 10%
10-24%
25-50%
>50%
I don't know

How do local pregnant patients who choose to give birth at another hospital that is further away for nonmedical reasons differ from those who give birth in your facility? [please select all that apply]
Insurance type
Financial resources
Access to transportation
Specific racial or ethnic group
Specific language or cultural group
Maternal age
Other, please explain below
No difference
I don't know

Display This Question:
If If How do local pregnant patients who choose to give birth at another hospital that is further away; for nonmedical reasons differ from those who give birth in your facility? QID31/SelectedChoicesCount Is Not Equal to 0
And How do local pregnant patients who choose to give birth at another hospital that is further away ... != No difference
And How do local pregnant patients who choose to give birth at another hospital that is further away ... != I don't know

Please explain or elaborate on how patients who bypass for nonmedical reasons differ from those who give birth in your facility.
____
____

How far away from your hospital is the closest Neonatal Intensive Care Unit (NICU)?
We have an on-site NICU
< 10 miles
10-29 miles
30- 60 miles
> 60 miles
I don't know

© 2022 Kozhimannil KB et al. *JAMA Health Forum.*

During hospital discharge after a live birth:

	Yes, almost always	Sometimes	Almost never or no	I don't know
Is the first postpartum appointment with their provider scheduled prior to discharge?	○	○	○	○
Do you specifically address cultural considerations in your postpartum planning?	○	○	○	○
Do you offer a follow-up call within a day or two after discharge?	○	○	○	○
Do you offer a home visit within a day or two after discharge?	○	○	○	○

Before COVID, were your labor and birth patients able to access telehealth/telemedicine services for: (select all that apply)
Prenatal visits
Postpartum visits
Well baby visits
Visits related to emergent concerns related to pregnancy or postpartum
Visits related to emergent concerns related to the newborn
No, telehealth/telemedicine was not available

In the COVID era, are your labor and birth patients able to access telehealth/telemedicine services for: (select all that apply)
Prenatal visits
Postpartum visits
Well baby visits
Visits related to emergent concerns related to pregnancy or postpartum
Visits related to emergent concerns related to the newborn
No, telehealth/telemedicine is not available

Section 2: Clinical Safety
This section will ask questions about the inpatient maternity care you provide at your hospital.

Are there any services, equipment, or facilities your hospital lacks that would help to provide safer care during labor and birth?
No
Yes, please explain ____

© 2022 Kozhimannil KB et al. *JAMA Health Forum.*

What support/training does your hospital offer to ensure that your staff have the skills and expertise necessary to provide safe care during labor and birth? (select all that apply)
In-services at the hospital
Online training/CME
Professional conferences
Travel to a different hospital or university for training
Simulations
Other, please explain ____
None

Please provide your most recent annual rates for the following:
Exclusive breastfeeding rate ____
Cesarean rate ____

Does your staff have the training and equipment to:

	Yes	No
Perform blood transfusions	○	○
Perform neonatal resuscitation	○	○
Provide emergency cesarean deliveries within one hour of determination of need	○	○

For cesarean deliveries, does your hospital use an operating room that is dedicated to obstetric cases or a general operating room that is also used for non-obstetric cases?
Dedicated operating room for obstetric cases
Main hospital/general operating room
We do not perform cesareans (please explain why) ____
Other, please explain ____

© 2022 Kozhimannil KB et al. *JAMA Health Forum.*

Before COVID, did your clinicians have **in person or telemedicine** support for consultations for:
(select all that apply)

	In person support	Telemedicine support	No consultation support
Obstetrics	☐	☐	☐
Maternal Fetal Medicine	☐	☐	☐
Pediatrics	☐	☐	☐
Neonatology	☐	☐	☐
Surgery	☐	☐	☐
Anesthesia	☐	☐	☐

In the COVID era, do your clinicians have **in person or telemedicine** support for consultations for:
(select all that apply)

	In person support	Telemedicine support	No consultation support
Obstetrics	☐	☐	☐
Maternal Fetal Medicine	☐	☐	☐
Pediatrics	☐	☐	☐
Neonatology	☐	☐	☐
Surgery	☐	☐	☐
Anesthesia	☐	☐	☐

© 2022 Kozhimannil KB et al. *JAMA Health Forum.*

Does your unit use resources to reduce maternal mortality and morbidity such as the CMQCC toolkits or the AIM safety bundles?
CMQCC toolkits [Mother & Baby Substance Exposure Initiative Toolkit, Improving Diagnosis and Treatment of Maternal Sepsis, Improving Health Care Response to Maternal Venous Thromboembolism, Improving Health Care Response to Cardiovascular Disease in Pregnancy and Postpartum, Toolkit to Support Vaginal Birth and Reduce Primary Cesareans, Improving Health Care Response to Obstetric Hemorrhage, Improving Health Care Response to Preeclampsia, Elimination of Non-medically Indicated (Elective) Deliveries Before 39 Weeks Gestational Age]
AIM patient safety bundles [Maternal Early Warning Signs (MEWS) Protocol for Obstetric Hemorrhage, Severe Hypertension in Pregnancy, Safe Reduction of Primary Cesarean Birth, Obstetric Care for Women with Opioid Use Disorder, Maternal Mental Health: Perinatal Depression and Anxiety, Maternal Venous Thromboembolism, Reduction of Peripartum Racial and Ethnic Disparities, Retained Vaginal Sponges After Birth, Support After Severe Maternal Event]
We do not use these resources.

Display This Question:
If Does your unit use resources to reduce maternal mortality and morbidity such as the CMQCC toolkit... = CMQCC toolkits [Mother & Baby Substance Exposure Initiative Toolkit, Improving Diagnosis and Treatment of Maternal Sepsis, Improving Health Care Response to Maternal Venous Thromboembolism, Improving Health Care Response to Cardiovascular Disease in Pregnancy and Postpartum, Toolkit to Support Vaginal Birth and Reduce Primary Cesareans, Improving Health Care Response to Obstetric Hemorrhage, Improving Health Care Response to Preeclampsia, Elimination of Non-medically Indicated (Elective) Deliveries Before 39 Weeks Gestational Age]
Or Does your unit use resources to reduce maternal mortality and morbidity such as the CMQCC toolkit... = AIM patient safety bundles [Maternal Early Warning Signs (MEWS) Protocol for Obstetric Hemorrhage, Severe Hypertension in Pregnancy, Safe Reduction of Primary Cesarean Birth, Obstetric Care for Women with Opioid Use Disorder, Maternal Mental Health: Perinatal Depression and Anxiety, Maternal Venous Thromboembolism, Reduction of Peripartum Racial and Ethnic Disparities, Retained Vaginal Sponges After Birth, Support After Severe Maternal Event]

Please describe your experience using the CMQCC toolkits or AIM safety bundles, including any barriers you encountered or facilitators to implementation.

____
____
____
____
____

Display This Question:
If Does your unit use resources to reduce maternal mortality and morbidity such as the CMQCC toolkit... = We do not use these resources.

Please describe any barriers you have experienced to implementing resources to prevent maternal mortality and morbidity such as the CMQCC toolkits or AIM safety bundles.

____
____
____
____
____

© 2022 Kozhimannil KB et al. *JAMA Health Forum.*

Section 3: Staffing
This section will ask questions about the maternity care clinicians and staff at your hospital.

Please tell us about the providers currently attending births at your hospital. (If you don't know an answer, please enter 9999)

	Number of clinicians
Obstetrician	____
Family practice doctor	____
Certified nurse midwife (CNM)	____
Other clinicians	____

How many full-time equivalents (FTEs) do you currently have for labor and birth unit RNs? (If you don't know, please enter 9999)

____

Does your hospital use any of the following scheduling techniques for registered nurses?
(select all that apply)
Cross training and working across other inpatient hospital units
Cross training and working in the hospital emergency department
Travel nurses
Cross training and working as the hospital's charge nurse
None of the above

Does your hospital use any of the following scheduling techniques for physician and midwife providers?
(select all that apply)
Laborist model
Locums providers
None of the above

What is your biggest challenge in staffing your labor and birth unit?

____
____
____
____
____

What works well in your labor and birth staffing in helping you to take good care of your patients?

____
____
____

Section 4: Financial Considerations
This section will ask questions about the financial portion of your hospital and obstetric service line.

Was your hospital operating in the black (revenues exceeding costs) in 2019?
Yes
No
Other, please explain ___________________________________

Was your obstetric service line/labor and birth unit operating in the black (revenues exceeding costs) in 2019?
Yes
No
Other, please explain ___________________________________

© 2022 Kozhimannil KB et al. *JAMA Health Forum.*

Approximately what percentage of the births at your hospital are paid for by:
(Note: The total should add up to 100%. Your best estimate is ok.)

	Percent
Medicaid	_____
Private or commercial insurance	_____
Self-pay or uninsured	_____
Indian Health Service	_____
Tricare	_____
Other	_____

Does your payor mix affect the minimum number of births necessary for financial viability?
Yes, please explain ____
No

Does your hospital offer services that reduce the fixed costs of providing your obstetric line? For example, general surgery services, which share anesthesia services, or ultrasound services in radiology?
Yes, please explain ____
No

Are you able to offer competitive clinician salaries that encourage retention and recruitment of obstetric personnel?
Yes ____
No ____
Other, please explain ____

Does your hospital offer funding and/or protected time to train labor and birth staff each year?
(select all that apply)

	Funds for training	Protected time for training	No training offered
Providers (obstetricians, midwives, family physicians)	☐	☐	☐
RNs	☐	☐	☐

Is the cost of medical liability insurance making it difficult to financially maintain the obstetric service line?
Yes, please explain ____
No

Are there other fixed costs making it difficult to financially maintain the obstetric service line?
Yes, please explain ____
No

Section 5: Wrap Up This is the final section of the survey. Thank you for sticking with us!

© 2022 Kozhimannil KB et al. *JAMA Health Forum.*

Please rank these factors regarding their influence on your decision to continue to provide inpatient labor and birth services. (most influential=1, least influential=4)
______ Local community needs
______ Clinical safety and training
______ Staffing, recruitment, and retention
______ Financial

Please explain your rankings above.

____

____

Assuming no changes in your community needs, workforce, or reimbursement, what is your prediction that you'll be providing inpatient labor and birth services at your hospital in 10 years?
Confident we will be offering inpatient labor and birth services
It is likely that our inpatient labor and birth services will close
Too difficult to predict at this time

Please explain your answer above.

____

____

Would you like us to email you a copy of the report of the survey results?
No
Yes, please provide your email here ____

Thank you very much for completing this survey. Is there anything else you want us to know about your hospital's labor and birth services or anything you'd like to clarify?

____

____

© 2022 Kozhimannil KB et al. *JAMA Health Forum.*

eTable 1: Comparing responding vs. non-responding hospitals among those with current obstetric services

Characteristic	Responded (n=93)	Not responded (n=199)	P-value*
**Financial structure**			
Critical Access Hospitals, n (%)	33 (35.5)	63 (31.7)	0.517
% of inpatient days that were Medicaid funded, median [IQR]	18 [12-23]	18 [12-24]	0.950
Hospital control, n (%)			0.736
Government, nonfederal	24 (25.8)	53 (26.6)	
Government, federal	2 (2.2)	9 (4.5)	
Nongovernment, not-for-profit	54 (58.1)	114 (57.3)	
For-profit	13 (33.3)	23 (11.6)	
**Location**			
County type, n (%)			0.908
Micropolitan	60 (64.5)	127 (63.8)	
Noncore	33 (35.5)	72 (36.2)	
Region, n (%)			0.052
Northeast	5 (5.4)	10 (5.0)	
Midwest	29 (31.2)	64 (32.2)	
South	26 (28.0)	82 (41.2)	
West	33 (35.5)	43 (21.6)	
County racial/ethnic majority, n (%)			0.556
Non-Hispanic white	64 (68.8)	130 (65.3)	
BIPOC or no majority	29 (31.2)	69 (34.7)	
**Hospital size**			
Average daily census, median [IQR]	22 [10-53]	20 [9-52]	0.958
**Obstetric services**			
No. of births in 2018 (via AHA), median [IQR]	311 [139-500]	343 [174-585]	0.335

*P-values are Chi-square or Wilcoxon rank-sum tests.

© 2022 Kozhimannil KB et al. *JAMA Health Forum.*

eTable 2: Descriptive information about survey respondents (n=93)

**Respondent characteristics**	**N (%)**
Highest level of education	
Some college	1 (1.1)
2 year degree	15 (16.1)
4 year degree	50 (53.7)
Masters	22 (23.7)
Doctorate	4 (4.3)
Doctor of Medicine (MD)	1 (1.1)
Years worked at current institution	
0-5 years	29 (31.2)
6-10 years	15 (16.1)
11-20 years	17 (18.3)
>20 years	32 (34.4)
Position or title	
Obstetric unit manager	69 (74.2)
Clinical leadership	12 (12.9)
Chief Nursing Officer	7 (7.5)
Administration	2 (2.1)
Chief Executive Officer	1 (1.1)
Chief Medical Officer	1 (1.1)
Head/Chief of Obstetrics	1 (1.1)

© 2022 Kozhimannil KB et al. *JAMA Health Forum.*
